# Paroxysmal Kinesigenic Dyskinesia With Infantile Convulsions in a Child With Inherited Co-deletion of 16p11.2 and 16p12.2: A Case Report and Literature Review

**DOI:** 10.7759/cureus.88761

**Published:** 2025-07-25

**Authors:** Man Ding, Bo Yin, Yin Liu, Jiajia Yao, Hongjuan Dong

**Affiliations:** 1 Department of Neurology, Renmin Hospital of Wuhan University, Wuhan, CHN

**Keywords:** 16p11.2, 16p12.2, paroxysmal kinesigenic dyskinesia, paroxysmal kinesigenic dyskinesia with infantile convulsions, prrt2

## Abstract

16p11.2 deletion syndrome is a group disorder associated with intellectual impairment, developmental delay, and autism spectrum disorder (ASD). Paroxysmal kinesigenic dyskinesia with infantile convulsions (PKD/IC) is an extremely rare condition. A 6-year-old Chinese boy presented with a two-month history of involuntary dystonic movements triggered by movement initiation (e.g., starting to walk, sudden activity after rest) or occurring spontaneously. He had a history of self-limiting infantile epilepsy from 6 months to 2 years of age. Notably, his father experienced benign infantile epilepsy during infancy (onset at 9 months, duration one year), and his grandparents were consanguineous. Based on clinical manifestations and whole-exome sequencing revealing biallelic inherited deletions, a 661.385 kb deletion at 16p11.2 (encompassing the PRRT2 gene) and a 760.266 kb deletion at 16p12.2 in the proband, with corresponding paternal deletions, the patient was diagnosed with PKD/IC. The patient received oral oxcarbazepine (OXC, 150 mg/day) to improve the symptoms. During follow-up after treatment initiation, his symptoms were well controlled, with no further PKD attacks. Early diagnosis of the disease is highly cost-effective and can help avoid unnecessary diagnostic and therapeutic interventions.

## Introduction

Paroxysmal kinesigenic dyskinesia (PKD), benign infantile epilepsy (BIE), and PKD with infantile convulsions (PKD/IC) are proline-rich transmembrane protein 2 (PRRT2)-associated paroxysmal disorders. PKD is characterized by brief attacks of kinesigenic involuntary movements. Convulsions or seizure attacks, designated as BIE, usually arise during the first year of life and commonly resolve by two years of age. PKD/IC is defined as the co-occurrence of both PKD and BIE.

The main features of 16p11.2 deletion are associated with multiple system abnormalities, including intellectual impairment, developmental delay, and features of autism spectrum disorder (ASD) [1]. Paroxysmal disorders are not frequently caused by 16p11.2 deletion. Deletions of 16p11.2, including PRRT2, are associated with 16p11.2 microdeletion syndrome (MIM 611913). PRRT2 mutations were first reported to be associated with PKD in 2011 [2], BIE and infantile convulsions with choreoathetosis syndrome (ICCA) in 2012 [3], and PKD/IC in 2012 [4]. A proband is diagnosed with PRRT2-associated paroxysmal disorders if they exhibit any of the following molecular genetic findings: a PRRT2 heterozygous pathogenic variant, a 16p11.2 recurrent deletion that includes PRRT2, or biallelic PRRT2 pathogenic variants (typically associated with a more severe phenotype). The incidence of 16p12.2 deletion is approximately 1:15,000 [5]. UQCRC2 (cytochrome b-c1 complex subunit 2) is the only Mendelian disease gene in this region in which mutations may cause epilepsy.

Here, we report a familial case of 16p11.2 and 16p12.2 co-deletion presenting with typical PKD/IC and BIE, and review the features of this syndrome.

## Case presentation

**Figure 1:**
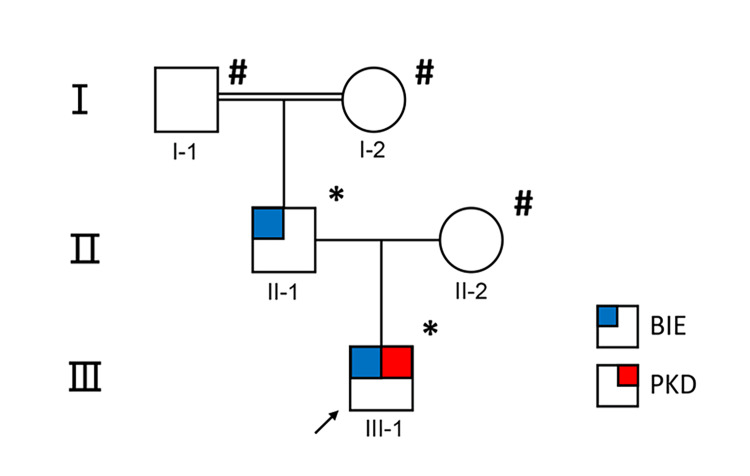
Pedigree structure of the familial case with PRRT2 variants. A double line indicates a consanguineous marriage between individuals I-1 and I-2. * Affected individuals were found to carry mutations within the 16p11.2 and 16p12.2 microdeletions based on mutation analysis; # Unaffected individuals from whom samples were obtained for mutation analysis. Arrows indicate the probands of the family. Empty circles and squares represent unaffected individuals. BIE: Upper left blue corner; PKD: Upper right red corner. BIE: Benign infantile epilepsy; PKD: Paroxysmal kinesigenic dyskinesia.

A 6-year-old boy presented with a 2-month history of involuntary dystonic movements. The episodes sometimes occurred when the patient attempted to start walking from a standing position, performed sudden activities after a period of physical rest, or occurred spontaneously without triggers. The attacks were unilateral, and the patient often attempted to suppress the movements using the unaffected side. The involuntary movements lasted approximately 10-15 seconds, with the patient’s right leg and hand not moving during the episodes. Attacks occurred with a frequency ranging from up to 10 times per day to once a week. The patient did not lose consciousness during these events. Other neurological functions, including speech, balance, and pain sensation, remained unaffected during the episodes. The patient first experienced an epileptic seizure at 6 months of age. Subsequently, seizures occurred approximately every 2-3 months. He was diagnosed with epilepsy and began antiepileptic drugs (AEDs), although the specific medication is unknown. Detailed information regarding the particular symptoms of the seizures was not recorded, and the family was unable to provide reliable descriptions. The seizures ceased after the age of 2 and have not recurred since. He was born with hypoxia as a low-birth-weight infant. However, his motor milestones and speech development were normal. There was no history of febrile convulsions. His mother’s previous pregnancy was normal, with an uncomplicated vaginal delivery, and she has no history of serious illnesses. The patient's father experienced seizures at 9 months of age, lasting for one year. These episodes, which occurred daily to monthly, often led to loss of awareness and limb convulsions. The specific medication used is unknown. There was no history of febrile convulsions or other neurological abnormalities in the father. The patient’s grandparents were consanguineous (Figure 1). Neurological and laboratory examinations of the patient, including routine serum tests, electrolytes, blood glucose, and ceruloplasmin, were within normal limits. Brain MRI and EEG results were unremarkable.

**Figure 2:**
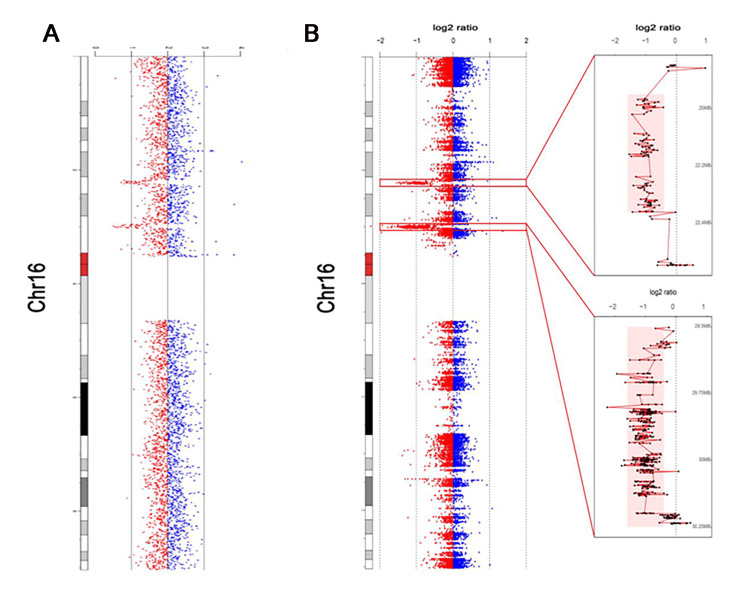
Copy number deletions in the familial case. (A) CNV analysis shows deletions at 16p11.2 and 16p12.2 in the proband. (B) Whole-exome sequencing (WES) results of chromosome 16 in the proband’s father. The identified 16p11.2 deletion is highlighted and enlarged in the chromosome view. CNV: Copy number variation.

Owing to the characteristics of sudden onset and abrupt cessation, paroxysmal disorders were suspected. Considering the family history, whole-exome sequencing (WES) and copy number variation (CNV) analysis were performed. The results confirmed inherited deletions of 661.385 kb on 16p11.2 (29539517-30200902) and 760.266 kb on 16p12.2 (21792450-22552716) in the proband (Figure 2A), and deletions of 16p11.2 (29606207-30199917) and 16p12.2 (21964686-22385666) in his father (Figure 2B). A diagnosis of PKD and BIE (referred to as PKD/IC) was made based on the clinical criteria (Appendix 1). Accordingly, the patient received oral administration of oxcarbazepine (OXC, 150 mg/day). During follow-up after treatment initiation, his symptoms were well controlled, with no further PKD attacks. 

## Discussion

**Figure 3:**
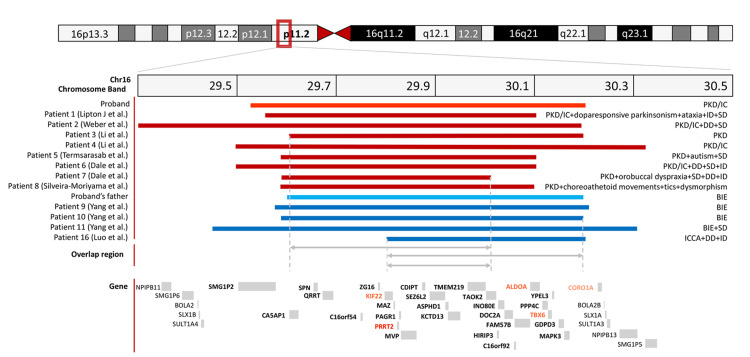
Genomic positions of the deletions and phenotypes of the PKD/PKD-IC and seizure disorder patients with 16p11.2 deletions. Genomic positions of the 16p11.2 deletions in patients with PKD or PKD/IC are shown as red bars, while those in patients with seizure disorders are shown as blue bars. Bright red and bright blue bars represent the 16p11.2 deletions identified in the present study. All other bars correspond to deletions reported in the literature. The associated phenotypes are listed on the right. The involved genes were mapped using the UCSC Genome Browser (http://genome.ucsc.edu/index.html, hg19 release) and are displayed below the bars. Mendelian disease genes are shown in red, with those identified in the present study highlighted in bright red. PKD: Paroxysmal Kinesigenic Dyskinesia; PKD/IC: Paroxysmal Kinesigenic Dyskinesia with Infantile Convulsions.

No cases of simultaneous 16p11.2 and 16p12.2 deletions have been reported. Clinically diagnosed PKD/IC in the proband and BIE in his father were determined according to the criteria summarized in Appendix 2. We speculate that the occurrence of PKD/IC and BIE is related to PRRT2, located in 16p11.2. Genetic testing revealed CNVs in 16p11.2 in two family members. The BP4 to BP5 region of 16p11.2 encompasses 29 genes and transcripts and five Mendelian disease genes (PRRT2, TBX6, ALDOA, KIF22, and CORO1A) (Figure 3). In addition, a 760 kb deletion (chr16:21792450-22552716) in the proband and a 420 kb deletion (chr16:21964686-22385666) in his father on 16p12.2 were also detected. The 16p12.2 deletion is associated with incomplete penetrance and variable expressivity, with a prevalence estimated at 1:15,000 [5]. The range of 16p12.2 includes ten genes and transcripts and one Mendelian disease gene (UQCRC2). Epilepsy may also occur in patients with UQCRC2 mutations; however, this does not adequately account for the PKD phenotype. Moreover, patients with TBX6, ALDOA, KIF22, or CORO1A mutations showed no manifestations of episodic disorders. PRRT2 is expressed throughout the CNS, with high levels detected in the cortical layers of the cerebral cortex and cerebellum (https://www.proteinatlas.org/ENSG00000167371-PRRT2/tissue). Mutations in PRRT2 have been identified in patients diagnosed with PKD/IC (OMIM #602066), PKD (OMIM #128200), and BIE (OMIM #605751). Among these CNVs and Mendelian disease genes in 16p11.2 and 16p12.2, CNVs in 16p11.2 that cover the PRRT2 gene are the most probable cause (Figure 3). To date, there have been no case reports of a 16p11.2 deletion accompanied by a 16p12.2 deletion co-occurring. It remains to be explored whether there is a relationship between these two deletions in one patient.

We systematically retrieved data from the PubMed database [6-16]. Fourteen patients with 16p11.2 deletions have been described with PKD or PKD/IC up to 2022 (Table 1, Figure 3). Including the proband, 51.7% of the patients (15/29; patients 1-14 and the proband) were diagnosed with PKD or PKD/IC. The age at PKD or PKD/IC onset ranged from 0 to 13 years (mean age 6y 8mo). Nine of the 15 (60.0%) were de novo mutations, two (13.3%, proband and patient 13) were inherited, and four (26.7%) were unknown (Table 1). The microdeletion size ranged from 432.033 kb to 894.907 kb. Except for patients 2, 7, and 9, for whom medication information was unavailable, all others used OXC (4/15, 26.7%) or carbamazepine (CBZ, 8/15, 53.3%). Patients 10, 11, 12, and 14 had incomplete OXC or CBZ control (4/15, 26.7%). Other neurological symptoms were also reported in patients of this cohort, such as dopa-responsive parkinsonism (patient 1), ataxia (patient 1), autism (patient 5), orobuccal dyspraxia (patient 7), choreoathetoid movements (patients 8, 9, 12, and 14), and tics (patient 8). Patient 3 had pure PKD with no other phenotypes or developmental disabilities. The proband and patient 4 presented with PKD/IC without other abnormalities. Nine patients (1, 2, 5, 6, 7, 10, 11, 12, and 13) exhibited speech delay (SD), including verbal, reading, or writing learning disabilities. Seven patients (1, 6, 7, 10, 11, 12, and 14) showed intellectual disability (ID). Patients 2, 6, 7, 12, and 13 presented with developmental delay (DD). Moreover, patient 8 showed subtle dysmorphism.

**Table 1:** Clinical and genetic details of PKD or PKD/IC with proximal 16p11.2 microdeletions. * sensitivity; † insensitivity; ‡ incomplete control aCGH: Array-based comparative genomic hybridization; BFIE: Benign familial infantile epilepsy; BIE: Benign infantile epilepsy; CBZ: Carbamazepine; CMA: Chromosomal microarray analysis; CNVs: Copy number variations; DD: Developmental delay; FS: Febrile seizure; IC: Infantile convulsions; ICCA: Infantile convulsions with choreoathetosis syndrome; ID: Intellectual disability; LEV: Levetiracetam; LTG: Lamotrigine; NA: Not available; NGS: Next-generation sequencing; OXC: Oxcarbazepine; P: Pathogenic; PKD: Paroxysmal kinesigenic dyskinesia; SD: Speech delay; SNP: Single nucleotide polymorphism; VPA: Valproic acid; WES: Whole-exome sequencing; ACMG: American College of Medical Genetics and Genomics.

Patient	Age at Onset (y.m), sex	Phenotypes	de novo/ inherited	Microdeletion Size (kb)	Deletion Coordinates	Treatment (dosage)	Developmental Disability	Chromosomal Microarray Analysis	History of Seizure	ACMG pathogencity	References	Year
Proband	0.6, M	PKD/IC	Inherited	661.385	chr16:29539517-30200902	OXC (300 mg/day)	No	CNVs	Yes, IC	P	Current case	
Patient 1	infantile, M	PKD/IC + doparesponsive parkinsonism + ataxia	de novo	544.342	chr16:29560500-30104842	CBZ (NA, very good to PKD); levodopa 200mg/day	SD + ID	SNP array	Yes, IC	P	Lipton J, Rivkin MJ [6]	2009
Patient 2	0, M	PKD/IC	NA	894.907	chr16:29303244-30198151	NA	SD + DD	SNP array	Yes, IC	P	Weber A et al. [7]	2013
Patient 3	11, M	PKD	de novo	591.997	chr16:29615090-30207087	CBZ (0.3 g/day)	No	NGS	No	P	Li W et al. [8]	2018
Patient 4	0.8, F	PKD/IC	de novo	832.76	chr16:29494072-30326832	OXC (450 mg/day)	No	NGS	Yes, IC	P	Li W et al. [8]	2018
Patient 5	10.5, M	PKD + autism	de novo	533.884	chr16:29564185-30098069	CBZ (200-250 mg/day)	SD	aCGH + SNP array	No	P	Termsarasab P et al. [9]	2014
Patient 6	0.11, M	PKD/IC	de novo	597.785	chr16:29500284-30098069	CBZ (400 mg/day)	Slight DD + slight SD + mild ID	Agilent aCGH 60K array	Yes, IC	P	Dale RC et al. [10]	2011
Patient 7	6, NA	PKD + orobuccal dyspraxia	de novo	432.033	chr16:29581455-30013488	NA	SD + DD + ID	CMA (agilent aCGH 60K array)	No	P	Dale RC et al. [11]	2012
Patient 8	5, M	PKD + choreoathetoid movements + tics	de novo	524.646	chr16:29581455-30106101	CBZ (400 mg/day)	Subtle dysmorphism	Microarray	No	P	Silveira-Moriyama L et al. [12]	2013
Patient 9	10, M	PKD+choreoathetoid movements	NA	627.7	chr16:29571443-30199160	NA	NA	CNV-seq	NA	P	Chen YL et al. [13]	2022
Patient 10	10, M	PKD	NA	627.7	chr16:29571443-30199160	OXC (150 mg/day) (±)	SD + ID	CNV-seq	No	P	Chen YL et al. [13]	2022
Patient 11	13, M	PKD	de novo	642.2	chr16:29557415-30199610	CBZ (100 mg/day) (±)	SD + ID	CNV-seq	No	P	Chen YL et al. [13]	2022
Patient 12	12, M	PKD/IC+choreoathetoid movements	NA	628.2	chr16:29571443-30199610	CBZ (50 mg/day) (±)	SD + DD + ID	CNV-seq	Yes, IC	P	Chen YL et al. [13]	2022
Patient 13	9, F	PKD/IC	Inherited	750.6	chr16:29449240-30199846	CBZ (50 mg/day) (+)	SD + DD	CNV-seq	Yes, IC	P	Chen YL et al. [13]	2022
Patient 14	13, M	PKD+choreoathetoid movements	de novo	524.8	chr16:29675050-30199897	OXC (150 mg/day) (±)	ID	CNV-seq	No	P	Chen YL et al. [13]	2022

Seizure disorders were also reported in this cohort, with a proportion of 48.3% (14/29) diagnosed with seizure disorders, whose onset age ranged from 0 to 15 years (mean age: 3y 3mo) (Table 2). None of them developed the classic features of PKD, whereas patients 22, 26, and 27 presented with ICCA. Focal epilepsy has been reported in patients 23 and 24. In addition, febrile seizures (FS) were observed in combination with choreoathetoid movements in patient 25. Among those for whom genetic information was available (patients 15, 16, 17, 22, 25, and 27), the mutations were de novo, with microdeletion sizes ranging from 397.816 kb to 863.087 kb (Table 2 and Figure 3). Only four patients (28.6%) in this cohort did not show DD, ID, or SD. The remaining patients (71.4%) had developmental disabilities. Additionally, 21.4% (3/14) of the patients received levetiracetam (LEV), among whom patients 16 and 17 showed poor treatment response. A total of 42.9% (6/14) received valproic acid (VPA) with sensitivity, while one patient (patient 21) switched to CBZ due to side effects. Patients 25 and 26 responded well to CBZ. Patient 27 received CBZ but continued to experience occasional attacks with incomplete control. Two patients received OXC (patient 22) and lamotrigine (patient 24); however, the effects of LTG remain unclear. The BIE, PKD, and PKD/IC caused by 16p11.2 microdeletion are often de novo but inherited in approximately 6.9% of cases, including the proband. An overview of these characteristics is presented in Table 3.

**Table 2:** Clinical and genetic details of seizure disorders with proximal 16p11.2 microdeletions. * sensitivity; † insensitivity; ‡ incomplete control aCGH: Array-based comparative genomic hybridization; BFIE: Benign familial infantile epilepsy; BIE: Benign infantile epilepsy; CBZ: Carbamazepine; CMA: Chromosomal microarray analysis; CNVs: Copy number variations; DD: Developmental delay; FS: Febrile seizure; IC: Infantile convulsions; ICCA: Infantile convulsions with choreoathetosis syndrome; ID: Intellectual disability; LEV: Levetiracetam; LTG: Lamotrigine; NA: Not available; NGS: Next-generation sequencing; OXC: Oxcarbazepine; P: Pathogenic; PKD: Paroxysmal kinesigenic dyskinesia; SD: Speech delay; SNP: Single nucleotide polymorphism; VPA: Valproic acid; WES: Whole-exome sequencing; ACMG: American College of Medical Genetics and Genomics.

Patient	Age at Onset (y.m), Sex	Phenotypes	de novo / Inherited	Microdeletion Size (kb)	Deletion Coordinates	Treatment (effect)	Developmental Disability	Chromosomal Microarray Analysis	History of Seizure	ACMG Pathogenicity	References	Year
Proband's father	0.9, M	BIE	de novo	593.71	chr16:29606207-30199917	NA	No	WES	Yes, IC	P	Current case	—
Patient 15	0.5, M	BIE	de novo	639.999	chr16:29571922-30211921	VPA (+)*	No	NA	Focal epilepsy	P	Yang L et al. [14]	2020
Patient 16	0.6, F	BIE	de novo	619.031	chr16:29580565-30199596	LEV (-)†	No	NA	Focal epilepsy	P	Yang L et al. [14]	2020
Patient 17	0.7, F	BIE	de novo	863.087	chr16:29455325-30318412	LEV (-); VPA (+)	SD (mild language delay)	NA	Focal epilepsy	P	Yang L et al. [14]	2020
Patient 18	0.4, F	BIE	NA	NA	NA	LEV (+)	DD	NA	No	P	Vlaskamp DR et al. [15]	2019
Patient 19	0.4, F	BIE	NA	NA	NA	VPA (+)	DD + ID	NA	No	P	Vlaskamp DR et al. [15]	2019
Patient 20	0.5, F	BIE	NA	NA	NA	NA	Severe DD + ID	NA	No	P	Vlaskamp DR et al. [15]	2019
Patient 21	0, F	BIE + myoclonic dystonia with cortical myoclonus	NA	NA	NA	VPA (+, but side effects); CBZ (+)	Motor DD	NA	No	P	Vlaskamp DR et al. [15]	2019
Patient 22	0.8, M	ICCA	de novo	397.816	chr16:29802081-30199897	OXC (+)	DD + ID	NGS	Yes, IC	P	Luo HY et al. [16]	2021
Patient 23	12, M	Focal epilepsy	NA	NA	NA	VPA (+)	DD + ID	NA	No	P	Vlaskamp DR et al. [15]	2019
Patient 24	0, F	Focal epilepsy	NA	NA	NA	VPA (+); LTG (unknown)	Severe DD + ID	NA	No	P	Vlaskamp DR et al. [15]	2019
Patient 25	11, M	Choreoathetoid movements + FS	de novo	617.8	chr16:29580569-30198321	CBZ (50 mg/day) (+)	SD + ID	CNV-seq	Yes, FS	P	Chen YL et al. [13]	2022
Patient 26	15, M	ICCA	NA	572.1	chr16:29557415-30129545	CBZ (100 mg/day) (+)	SD + ID	CNV-seq	Yes, IC	P	Chen YL et al. [13]	2022
Patient 27	4, M	ICCA	de novo	642.2	chr16:29557415-30199610	CBZ (100 mg/day) (±)	No	CNV-seq	Yes, IC	P	Chen YL et al. [13]	2022

**Table 3:** Characteristics of 16p11.2 microdeletions. BIE: Benign infantile epilepsy; CBZ: Carbamazepine; IC: Infantile convulsions; ICCA: Infantile convulsions, choreoathetosis syndrome; LEV: Levetiracetam; LTG: Lamotrigine; NA: Not available; OXC: Oxcarbazepine; PKD: Paroxysmal kinesigenic dyskinesia; VPA: Valproic acid.

Characteristics	n	%
Phenotypes		
PKD	8	27.59%
PKD/IC	7	24.14%
BIE	8	27.59%
Others	0	0.00%
Dopa-responsive parkinsonism	1	3.45%
Ataxia	1	3.45%
Autism	1	3.45%
Orobuccal dyspraxia	1	3.45%
Choreoathetoid movements	5	17.24%
Tics	1	3.45%
Myoclonic dystonia with cortical myoclonus	1	3.45%
ICCA	3	10.34%
Focal epilepsy	2	6.90%
Febrile seizure	1	3.45%
Speech delay	12	41.38%
Intellectual disability	14	48.28%
Developmental delay	12	41.38%
Dysmorphism	1	3.45%
Mutations		
de novo (PKD & PKD/IC, Seizure)	16 (9, 7)	55.17%
Inherited (PKD & PKD/IC, Seizure)	2 (2, 0)	6.90%
NA	11	37.93%
Treatments		
OXC	5	17.24%
CBZ	11	37.93%
VPA	6	20.69%
LEV	3	10.34%
LTG	1	3.45%
Levodopa	1	3.45%
NA	5	17.24%

Among the 29 patients, the CNV sizes for PKD and epilepsy/convulsions were ≥430 kb and 390 kb, respectively. The median sizes were similar (627.663 kb vs. 618.218 kb, respectively). The overlapping region of PKD in the proband and other patients ranged from 29675050 to 30013488, whereas the overlapping region of BIE ranged from 29802081 to 30129545. The common overlapping region between PKD and BIE was 211.407 kb (29802081 to 30013488) (Figure 3). No evidence verified significant differences in deletion size between BIE and PKD or PKD/IC. In contrast, PKD or PKD/IC are more likely to involve the anterior part of the common region of chromosome bands, while BIE is more likely to affect the posterior part.

Once PRRT2 pathogenic variants are identified in an affected family member, prenatal testing and preimplantation genetic testing are possible for pregnancies at increased risk; however, reduced penetrance and clinical heterogeneity may lead to phenotypic variations within families. When family history and phenotype suggest a diagnosis of PKD or PKD/IC, single-gene testing or a multigene panel can be selected as molecular genetic testing methods. However, for the diagnosis of PKD or PKD/IC not clinically suspected, comprehensive genomic testing, including exome sequencing and genome sequencing, may be a better diagnostic approach.

The rarity of this specific co-deletion makes definitive conclusions challenging. However, the presence of the 16p12.2 deletion in this familial case presenting with PKD/IC provides a unique opportunity for future research. Several issues remain to be explored: (a) Whether there is an association between the co-deletion of 16p12.2 and the onset time, severity of the condition, or treatment response compared to isolated 16p11.2 deletions or PRRT2 point mutations; (b) Whether there are potential interactions between genes within the 16p12.2 region (such as UQCRC2, CACNA1H) and PRRT2, leading to gene dosage or interaction effects; (c) Whether 16p12.2 deletions act as genetic modifier factors, thereby influencing the phenotypic spectrum and prognosis of PRRT2 mutations.

## Conclusions

Finally, we propose that rapid genetic analysis in the early stages of the disease is highly cost-effective and can help avoid unnecessary diagnostic and therapeutic interventions. In co-deletion carriers, asymptomatic parents may transmit pathogenic CNVs, and multi-locus CNVs may underlie complex paroxysmal phenotypes previously attributed to single-gene defects. Future studies should explore whether 16p12.2 deletions act as phenotypic modifiers in PRRT2-related disorders.

## Appendices

Appendix 1

**Table 4:** Criteria for the diagnosis of PKD, BIE, and PKD/IC. PKD: Paroxysmal kinesigenic dyskinesia; BIE: Benign infantile epilepsy; PKD/IC: Paroxysmal kinesigenic dyskinesia with infantile convulsions

Diagnosis	Clinical Findings	Supportive Findings
Paroxysmal Kinesigenic Dyskinesia (PKD)	1. Identification of episodic kinesigenic triggers	1. Normal neurologic examination between attacks
	2. Short duration of attacks (<1 minute)	2. Normal brain MRI
	3. No loss of consciousness or pain during episodes	3. No EEG changes during attacks
	4. Exclusion of other organic diseases and normal neurological findings in the case of primary PKD	-
	5. Control of episodes with phenytoin or carbamazepine, if attempted	-
	6. Age of onset between 1 and 20 years in cases with a negative family history of PKD	-
	Note: Molecular genetic screening for the PRRT2 gene may be useful to confirm the diagnosis.	-
Benign Infantile Epilepsy (BIE)	1. Onset in the first year of life (most commonly between 4–12 months)	1. Otherwise normal development and outcome
	2. Occurrence either spontaneously or in the context of fever	2. Normal neurologic examination
	3. May occur in clusters of multiple seizures per day (up to 8–10 seizures/day every 2–3 hours)	3. Normal brain imaging and EEG background signal
	4. Excellent response to antiepileptic drugs (AEDs)	-
	5. Resolution by age two years	-
Paroxysmal Kinesigenic Dyskinesia with Infantile Convulsions (PKD/IC)	Must meet both:	-
	1. Paroxysmal movement disorder that meets criteria for PKD	-
	2. Seizures that meet criteria for BIE	-

Appendix 2 

**Table 5:** Main Mendelian disease genes in 16p11.2 and 16p12.2. AD: Autosomal Dominant; AR: Autosomal Recessive; MIM: Mendelian Inheritance in Man.

Chromosome	Gene	Protein	Phenotype (Inheritance)	MIM Number
16p11.2	PRRT2	Proline-rich transmembrane protein 2	Convulsions, familial infantile, with paroxysmal choreoathetosis (Autosomal Dominant)	602066
			Episodic kinesigenic dyskinesia 1 (Autosomal Dominant)	128200
			Seizures, benign familial infantile, 2 (Autosomal Dominant)	605751
	TBX6	T-box transcription factor 6	Spondylocostal dysostosis 5 (Autosomal Dominant, Autosomal Recessive)	122600
	ALDOA	Fructose-bisphosphate aldolase A	Glycogen storage disease XII (Autosomal Recessive)	611881
	KIF22	Kinesin-like protein 22	Spondyloepimetaphyseal dysplasia with joint laxity, type 2 (Autosomal Dominant)	603546
	CORO1A	Coronin-1A	Immunodeficiency 8 (Autosomal Recessive)	615401
16p12.2	UQCRC2	Cytochrome b-c1 complex subunit 2	Mitochondrial complex III deficiency, nuclear type 5 (Autosomal Recessive)	615160
